# Natural Biopolymers as Smart Coating Materials of Mesoporous Silica Nanoparticles for Drug Delivery

**DOI:** 10.3390/pharmaceutics15020447

**Published:** 2023-01-29

**Authors:** Bianca Dumontel, Verónica Conejo-Rodríguez, María Vallet-Regí, Miguel Manzano

**Affiliations:** 1 Department of Chemistry in Pharmaceutical Sciences, School of Pharmacy, Universidad Complutense de Madrid, UCM, Research Institute Hospital 12 de Octubre, imas12, 28040 Madrid, Spain; 2 Networking Research Center on Bioengineering, Biomaterials and Nanomedicine (CIBER-BBN), 28029 Madrid, Spain

**Keywords:** nanomedicine, smart nanotechnology, nanoparticles for drug delivery system, biopolymers coating, endogenous-stimuli, controlled release

## Abstract

In recent years, the functionalization of mesoporous silica nanoparticles (MSNs) with different types of responsive pore gatekeepers have shown great potential for the formulation of drug delivery systems (DDS) with minimal premature leakage and site-specific controlled release. New nanotechnological approaches have been developed with the objective of utilizing natural biopolymers as smart materials in drug delivery applications. Natural biopolymers are sensitive to various physicochemical and biological stimuli and are endowed with intrinsic biodegradability, biocompatibility, and low immunogenicity. Their use as biocompatible smart coatings has extensively been investigated in the last few years. This review summarizes the MSNs coating procedures with natural polysaccharides and protein-based biopolymers, focusing on their application as responsive materials to endogenous stimuli. Biopolymer-coated MSNs, which conjugate the nanocarrier features of mesoporous silica with the biocompatibility and controlled delivery provided by natural coatings, have shown promising therapeutic outcomes and the potential to emerge as valuable candidates for the selective treatment of various diseases.

## 1. Introduction

The application of nanotechnology to medicine has given rise to the birth of a new discipline: Nanomedicine, which is a multidisciplinary field with many key players, such as, chemists, biologists, physicians, engineers, physicists, and even legislators [1]. Nanomedicines have become very popular among the scientific community because of a number of factors, such as the control over the pharmacokinetic profile; the protection of the transported therapeutic agents against possible degradation; the possibility of developing therapies targeted towards specific tissues; the possibility of including different therapeutic agents in the same carrier; and even the possible inclusion of contrast agents to have a biomedical image useful in diagnosis. Among the available drug delivery nanosystems, nanoparticles have called attention to the scientific community since they are highly versatile from the point of view of composition, shape, size, and outer surface [2]. This makes them the focus of a great deal of biomedical research, whether for the treatment of certain diseases, prevention, diagnosis, or even tissue engineering.

Research on nanoparticles for drug delivery has been increased in the last few years due to several factors, such as their potential to improve stability and solubility of encapsulated cargos, their potential to cross biological membranes, and their capacity to prolong circulation times to therefore increase safety and efficacy [3]. Starting with liposomes, many types of nanoparticles have been investigated as effective drug delivery systems (DDS) [4,5]. Among others, mesoporous silica nanoparticles (MSNs) present attractive structural and textural properties, which allow the loading of large amount of therapeutic molecules, fulfilling one of the fundamental requirements for an effective nanocarrier [6]. However, their open pore structure also hinders the control over specific release and the premature leakage of therapeutic payloads constitutes one of the main challenges for MSNs application. 

Thereby, surface modification with functionalizing agents able to regulate the opening of MSNs pores is of prominent interest [7,8]. Smart capping agents can provide a barrier against premature release while ensuring on-demand release triggered by selected stimuli. This functionalization strategy thus constitutes a valuable approach to minimize off-target side effects and guarantee a selective delivery only in interested pathological areas. 

**Scheme 1:**
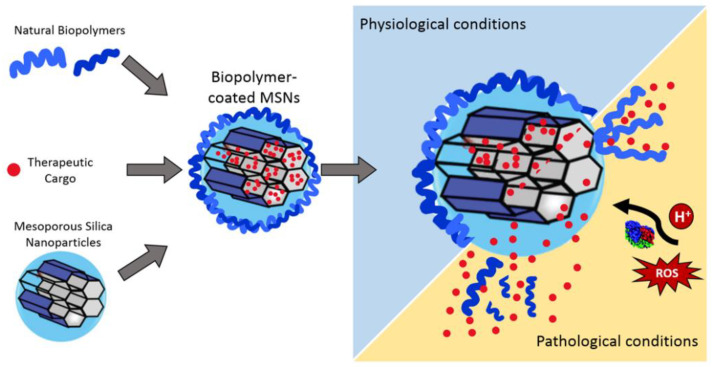
Graphical representation of the concept of coating Mesoporous Silica Nanoparticles (MSNs) to be employed as stimuli-responsive nanocarriers.

The research of gatekeepers with required sensitivity and biocompatibility is continuously ongoing and, in this scenario, this review will cover the application of biopolymers as novel coating agents for the formulation of stimuli-responsive DDS that combine the properties of MSNs as nanocarrier with the biocompatibility, non-immunogenicity, and innate sensitivity to endogenous stimuli of natural biopolymers (Scheme 1).

## 2. Mesoporous Silica Nanoparticles

### 2.1. Brief Description about the Origin, Development, and Classification of MSNs

Ordered mesoporous silicas were initially developed by a research team in Japan [9] and scientists at Mobil Oil Corp [10]. These mesoporous materials were based on the use of surfactants to produce a porous mesostructure with several applications in the field of catalysis and adsorbents. The first time that those silicas were explored as drug delivery matrices was presented by Vallet-Regí et al. in a seminal paper that started a broad scientific area such as the biomedical applications of ordered mesoporous silicas [11]. This extremely popular new field presents very high current activity with thousands of publications appearing every year [12]. The reasons behind such popularity come from the number of features of ordered mesoporous materials that make them suitable for drug delivery technologies, such as their ordered porous network that allows an accurate control of the cargo load and release kinetics; their large pore volume that allows hosting many different therapeutic agents; their huge surface area that guarantees an elevated amount of molecules adsorbed within the matrices; and their silanol-based surface, that enables easy functionalization reactions leading to great control over the drug loading and release processes [13]. Those unique physicochemical properties fueled the rapid translation from bulk to the nanoscale dimension [14]. The synthesis of these Mesoporous Silica Nanoparticles, a term coined by Victor Lin to illustrate nanoparticles made of mesoporous silica with a well-defined and controllable morphology, is based on the combination of three different methods. The three-dimensional network of silica is produced through the sol-gel process, where the hydrolysis and condensation of the selected silica precursors takes place. This is accompanied by the presence of surfactants as structure directing agents during the condensation step, and the subsequent removal of the template. 

Carrier nanoparticles for drug delivery purposes need to be in the range between 10 nm and few hundred nanometers. In fact, literature studies have observed that sub-6 nm nanoparticles or biodegradable particles can be easily excreted by the body through the kidneys, while sub-15 nm particles are capable of crossing the blood–brain barrier (BBB), and sub-200 nm nanoparticles are preferentially accumulated in cancer cells mainly exploiting the altered vasculature of tumoral areas [15]. Therefore, conventional synthetic protocols for bulk mesoporous materials were adapted through some modifications to the Stöber method to develop spherical monodisperse silica nanoparticles [16,17,18,19]. The synthesis of MSNs is conventionally carried out at low surfactant concentrations The manipulation of many reaction parameters, such as the pH, temperature, surfactant type, and concentration, etc., might result in MSNs with a variety of different morphologies, dimensions, pore sizes, and structures [20,21].

### 2.2. MSNs Properties for Drug Delivery Applications

Some of the most important features of a drug delivery nanosystem include the control both on the cargo loading and release rate processes of the transported drug, together with the specific delivery to a target tissue or cell [22]. There are many studies focused on numerous types of nanoparticles developed for drug delivery purposes, such as liposomes and lipid nanoparticles, polymeric nanoparticles, or inorganic nanocarriers [2]. Among them, mesoporous silica nanoparticles have called the attention of the biomedical research community due to their outstanding properties for controlled drug delivery purposes. The most significant features for that biomedical application are: their structural properties, such as ordered pore structure; their textural properties, such as narrow pore size distributions, large volumes, and surface areas; and their chemical properties, such as their surface full of silanol groups that ease the covalent grafting of many different organic groups [6]. 

The structural properties of ordered mesoporous materials in general, and MSNs in particular, are a consequence of the synthetic method in which surfactants act as templates for the condensation of the silica. In fact, the use of surfactants to direct the silica precursors condensation is a key step in the synthetic process, and many different surfactants have been employed for the creation of ordered mesoporous silicas [23]. Then, the removal of the surfactant, which can be carried out through a plethora of methods, leads to a network of cavities where the drug molecules can be loaded. Consequently, the ordered and reproducible mesostructures produced would guarantee the reproducibility of the cargo loading and release processes. This reproducible behavior would be of highly interest in the future translation of these nanomedicines to the clinic [24].

The textural properties, including pore diameter, pore volume, and surface area, are of capital importance for the efficiency of MSNs as drug delivery systems. The pore diameter acts as a limiting factor in terms of size of the type of drug that can be adsorbed. Therefore, it should be adapted to the size of the therapeutic agent to be introduced into the pores. Additionally, the pore diameter can also act as a regulator of the release kinetics, since it could limit the diffusion of the cargo molecules into the surrounding environment [25]. The pore volume plays an important role in the amount of drug molecules than can be loaded, since the larger the available volume, the greater the amount of pharmaceutical agents loaded. Additionally, the drug–drug interactions might help to increase the molecules retained within the mesoporous cavities [26]. Similarly, the surface area has always been a key parameter to adsorb molecules, since this is a physical interaction with the surface of the material. Therefore, the larger the surface area, the greater is the number of adsorbed molecules in their surface, and ordered mesoporous materials are characterized by their enormous surface area values [27].

The chemical properties of MSNs are conditioned by the high density of silanol groups on their surface. Consequently, it is very easy to use those silanol groups for functionalization with a variety of organic groups. This relatively easy process allows modification of the surface of the matrices, both internal and external, providing the MSNs with an almost infinite number of different functionalities that would control the behavior of the nanocarriers, as shown below. This is of capital importance in drug delivery applications, since it makes it possible to control the adsorption and release of the cargo molecules [13,28]. 

### 2.3. MSNs for Drug Delivery Applications

Regardless of the type of nanocarrier employed, any nanoparticle designed as a drug delivery system should be able to carry the cargo to certain locations of the body, increasing the efficacy of the transported drug and reducing any potential side effects. Therefore, these nanocarriers should be designed with special attention to their biological behavior, such as biocompatibility, biodistribution, biodegradability, or the potential clearance by the body. In fact, these parameters (adsorption, distribution, metabolism, and elimination) are some of the most important prerequisites for any nanomedicine to be used in the clinic [29]. In this sense, even though MSNs are well known to be chemically, thermally, and mechanically stable, they are degradable under physiological conditions [30,31]. Many different parameters have been identified to influence on the degradation of MSNs, such as nanoparticle morphology, size and concentration, pore size and surface area, condensation degree, any potential doping, surface modification, and the physiological medium [31,32]. Their degradation in different animal models, that is, their biodegradation, has been observed to strongly depend on their chemical composition. Thus, the surface functionalization of MSNs with certain polymers improved their in vivo stability, and therefore increased their blood circulation half-life, which is of capital importance when targeted as drug delivery systems [33,34,35,36,37,38,39,40,41,42,43,44]. Additionally, particle size and surface have also been found to present a strong influence on the biodistribution of MSNs, with different bloodstream half-lives depending on the particle [45,46]. 

Overall, the biocompatibility of MSNs has been observed to depend on some of their physicochemical properties. Consequently, MSNs should be designed according to their final application so good biocompatibility, low toxicity and predicted degradation, and clearance could be ensured [12].

## 3. Engineering of MSNs as Stimuli-Responsive Drug Delivery Systems

### 3.1. Types of MSNs Gatekeepers 

Due to their structural and mechanical properties, MSNs are able to load high amounts of therapeutic molecules, increasing the stability of cargos and preventing their degradation in biological environment [12]. However, pristine MSNs are not able to guarantee specific delivery, and the premature release of therapeutic payloads is considered one of the major shortcomings for their effective clinical translation [47]. For this reason, strategies involving the engineering of MSNs surface with responsive pore-blocking agents are widely investigated. Indeed, opportunely selected moieties can act as gatekeepers, regulating the closing or the opening of the pores in response to precise stimuli. In principle, this approach will allow to seal the MSNs pores in physiological conditions minimizing premature drug leakage and off-sites effects, while providing on-demand release in pathological area under specific triggers. The first approach was reported in 2003 by Mal et al. when developing coumarin-modified MSNs for the controlled release of a steroid precursor under UV-irradiation [48]. Since then, many types of gatekeepers and functionalization strategies have been implemented to design stimuli-responsive MSNs for different drug delivery applications [7,47,49]. Based on their composition, smart coatings can be divided into inorganic coatings, including inorganic nanoparticles or coating layers and organic coatings made by various types of linear molecules, macrocyclic compounds, and biomacromolecules.

Inorganic metal, metal-oxide, and graphene oxide nanoparticles or quantum dots conjugated to the silica surface have been used as capping agents for controlled release, triggered by the nanoparticles decomposition or by the rupture of chemical links [50,51,52]. The selection of nanoparticles with intrinsic cytotoxicity [53,54] or with luminescent [55] and magnetic [56,57] properties also allowed the introduction of additional features into functionalized MSNs by supporting the therapeutic action of released drugs or introducing imaging capabilities, respectively. 

More often, different kind of organic materials are used as MSNs gatekeepers. Among them, synthetic polymers have been the object of extensive studies due to their chemical variety, which allow their application in combination with different biological internal stimuli or physical stimuli applied from outside the organism [58,59,60,61]. The widespread application of polymeric gatekeepers is also connected with the possibility to tailor their composition and obtain materials with partial biodegradability and reduced immunogenicity. Some classes of synthetic polymers, such as polyesters or polyethers, show abilities for stimuli-responsive release together with good biocompatibility profiles [62,63,64,65]. Therefore, they have been largely applied as MSN capping agents and are often classified as biopolymers. 

**Figure 1:**
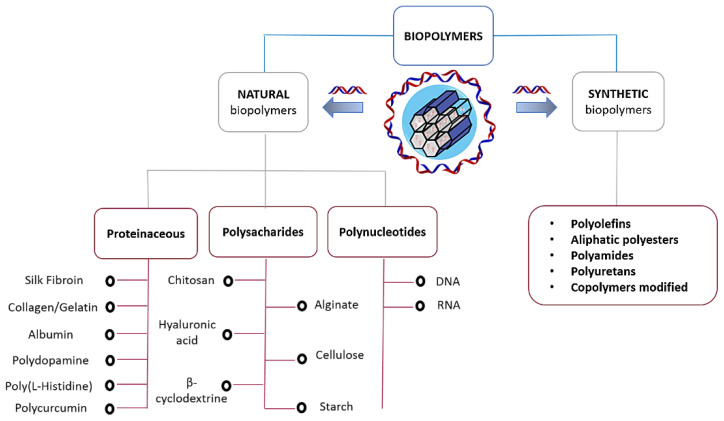
General classification of biopolymers used as capping agents for stimuli-responsive MSNs.

The prefix ‘bio’ is employed to highlight the biodegradability of the materials and the term biopolymers refers both to biodegradable polymers obtained by chemical polymerization processes (synthetic biopolymers) and to polymers with natural origins (natural biopolymers), as schematized in Figure 1.

Natural biopolymers are high molecular weight compounds biosynthesized by living organisms during metabolic processes and made by sequences of covalently bonded repeating units [66]. According to the repeating units, they are normally classified as polynucleotides, polypeptides/proteins, and polysaccharides (Figure 1). As metabolic products, natural biopolymers can be extracted from a variety of natural sources such as plants, seaweeds, animals, and microorganisms. Due to their natural derivation, natural biopolymers are highly sustainable materials with excellent biocompatibility and biodegradability and are therefore gaining increasing attention over synthetic ones in the field of drug delivery [67,68]. 

### 3.2. External Stimuli and Internal Stimuli

The research of appropriate gatekeepers is closely related to the knowledge of the stimuli that are naturally present or might be applied at the specific pathological site [69,70]. This knowledge allows the selection of moieties with suitable response for the desired therapeutic outcomes. Therefore, some of the key aspects of different stimuli applied to trigger the release of therapeutic cargo from engineered MSNs will be here summarized. In general, stimuli are classified as external or internal stimuli depending on whether they are applied from outside or inside the body, respectively [7].

#### 3.2.1. External Stimuli

External stimuli include temperature, light, electric and magnetic fields, or ultrasounds. They can be applied remotely from outside the organism and can be turned on/off as required, enabling pulsatile responsive release from activated nanocarriers and highly precise treatment of the disease [71].

- Temperature
  The overexpressed inflammatory markers in the infection or inflammation processes and tumors tissues can provoke moderate temperature increases of up to 4 or 5 °C as an immune system response by the leukocytes of the organism. Thermosensitive gatekeepers in nature grafted to MSNs have been used to block the pores entrances and avoid the diffusion of payload in an unspecific site as well as for displaying a lower critical solution temperature (LCST) at 37 °C. In this sense, the water-soluble polymers are ideal candidates to apply this kind of external stimuli because they are able to respond to temperature changes since they suffer a reversible conformational change in response to temperature variations over LCST. Another benefit is to enhance the colloidal stability of the MSNs. The most commonly employed are poly(*N*-isopropylacrylamide) (pNIPAM) and several analogs, as well as polyethylene glycol acrylates (PEG acrylates) or natural biopolymers agar and agarose, since they suffer a degradation or alteration of the polymer network that provokes drug releases as a consequence of induced localized hyperthermia [72,73,74,75,76,77,78].
- Magnetic stimuli
  It has been employed for triggering drug release from MSNs through the application of permanent or alternating magnetic fields that provoke an increase of temperature, exploited to generate hyperthermia-mediated cell death in different biomedical applications [79,80,81,82]. In this context, superparamagnetic iron oxide nanoparticles (SPIONs) are the most widely applied for magnetic stimuli-responsive drug delivery due to their capacity to transform magnetic energy into heat through Brownian or Nells fluctuations [83]. Normally these nanoparticles, for example Fe_3_O_4_ NPs, are encapsulated into MSNs using aerosol techniques or sol-gel processes [79,84,85].
- Light
  This type of exogenous stimulus can include and select different regions of the wavelengths as ultraviolet, visible and near-infrared light [86,87] and it has been explored by many researchers as a non-invasive method and spatiotemporal control for triggering drug release from MSNs [88,89,90,91]. Its easy application, low toxicity, and precise focalization constitute some of the advantages, while low tissue penetration is the major drawback.
- Ultrasound
  This external stimulus presents an easy regulation of tissue penetration depth by tuning some basic parameters, absence of ionizing radiations and non-invasiveness. For these reasons, it is an efficient element for carrying out cargo delivery at the target site with spatiotemporal control from responsive MSNs without any damage of the healthy tissues [59,63,92,93,94].

#### 3.2.2. Internal Stimuli

Internal stimuli, also named endogenous stimuli, includes pH, redox potential and enzymes, among other chemical variations that take place between normal and diseased tissues in the human body. The pathology usually provokes the overexpression or downregulation of certain relevant biomarkers which can be used to trigger the release of therapeutic cargos from MSNs by choosing the right moiety, namely sensitive linker and/or capping agent, capable of reacting to biochemical and metabolic processes involved in the targeted pathology.

- pH
  pH has become the focus of numerous investigations in oncology and the engineering of MSNs with gatekeepers responsive to the lower extracellular pH of tumor and inflamed tissues has been a common strategy for the targeted release of anticancer therapeutic agents. The cancerous or malignant cells produce acidic byproducts during their altered metabolic behavior that are transported to the extracellular environment producing a tumor microenvironment with pH between 6.0 and 7.0, while extracellular pH of normal tissues is 7.4 (standard physiological pH). In a similar way, during the endocytosis process, internalized nanoparticles are exposed to a pH-gradient depending on the cell compartment or organelle. Intracellular organelles, such as endosomes (pH = 5.5) and lysosomes (pH < 5.5) also have an acidic pH [95,96,97,98]. Both phenomena have been widely exploited by researchers to design novel nanocarriers based on biopolymer coated-MSNs able to deliver the therapeutic cargo entrapped in the mesopores by the chemical shift in the selected pH-sensitive gatekeeper molecules employed. This review will describe in detail some examples of nanosystems with sensitive-pH proteinaceous and polysaccharides biopolymers as coating and capping agents.
- Enzymes
  Numerous pathological states or diseased tissues provide the dysregulation of certain enzymes and/or specific antibodies, both hypo- or overexpressed. In fact, proliferative or metastatic behavior are often stimulated by enzymes overexpressed from malignant cells. These enzymes can be found in either an extracellular or intracellular environment, giving rise to additional ways for gatekeeper application. As a result, this mechanism can also be used for triggering the drug release on-demand from MSNs with very high specificity, accuracy, and efficiency, since enzymes have the ability to cleave very specific peptidic sequences [99,100,101,102].
  It is important to note the role of the matrix metalloproteinases (MMPs), especially MMP2, as one of the important internal pathological changes of the tumor microenvironment. They are overexpressed in almost every type of human cancer and associated with tumor invasiveness, metastasis, and angiogenesis [103]. Matrix metalloproteinases are enzymes responsible for remodeling the extracellular matrix (ECM) and for that reason they are capable of degrading all kinds of ECM proteins, including many biopolymers, playing a key role in angiogenesis and metastasis. Thus, their efficient catalytic capability is especially appealing to prepare tailor-made nanodevices with enzymatic response. This revision provides representative examples of smart nanodevices consisting of MSNs end-capped via enzyme-degradable biopolymers.
- Redox potential
  Distinct concentration of certain reductive species, such as glutathione (GSH) and reductive oxygen species (ROS), between the intra-cellular and the extra-cellular space, and also between healthy and tumor tissues, represent another interesting approach to develop smart drug delivery nanocarriers [99,104,105]. For instance, the overexpression of redox species such as GSH, which can be found four times higher in tumors than healthy tissues, is employed to design redox-responsive MSNs [106]. This kind of molecule is able to cleave different capping agents such as biopolymers grafted to MSNs via disulfide (S–S) bonds. Examples of this methods can be found bellow.
- Small molecules
  Similarly, certain chemical species are produced or accumulated in unbalanced amounts by diseased tissues. Gatekeepers sensitive to these types of molecules have been employed to cap MSNs and control the drug release outpointing glucose, antigens, and adenosine triphosphate (ATP)-aptamers, among others, as the most important biogenic biomolecules applied as triggers in the literature [107,108,109,110].

### 3.3. Advantages of Natural Biopolymers as Smart Coating Material

The use of biopolymers as capping agents forms part of a recent trend that envisages the functionalization of synthetic nanocarriers with biologically derived materials to enhance DDS safety [47,111]. Indeed, natural biopolymers present a composition similar to that of biological components of the extracellular matrix, and several studies reported their capability to avoid immunogenic responses and their low toxicity [47,112,113], which is also able to enhance the cytocompatibility of coated inorganic or polymeric nanoparticles [114,115,116]. Several natural biopolymers exhibit a hydrophilic nature, which can prevent protein adsorption and reduce opsonization process [117], making them valuable, natural alternatives to PEG to prolong the circulation of coated nanocarriers [118,119,120]. Moreover, natural biopolymers can be fully biodegraded in non-toxic metabolic products and this represents another great advantage compared to their synthetic counterparts [121]. The high biodegradability of natural biopolymers contributes not only to their biocompatibility, but also to their innate stimuli-responsive behavior and particular sensitivity to internal triggers. Their biological degradation, catalyzed by specific enzymes and oxidizing molecules, can be conveniently used to trigger the release from biopolymer-based DDS in pathological areas, which are characterized by altered metabolic behaviors and overexpress these agents.

Natural biopolymers possess hydroxyl, amino, or carboxyl functional groups in their structure, which ensure their good chemical reactivity and a versatility similar to synthetic polymers. For instance, these reactive groups enable modifications for the enhancement of biopolymer stability in biological environments and derivatives with improved solubility and have been largely investigated [122,123,124]. Additionally, crosslinking strategies with PEG [125,126], aldehydes [81,127], genipin, and other natural or synthetic crosslinkers [128] have been exploited to improve the biological stability and prevent the fast degradation of different natural biopolymeric coatings. 

Biopolymer reactive groups also play a central role in DDS formulation and performances. They are the mainly responsible for the structural and chemical changes exploited to trigger cargo release in response to pH or redox stimuli. Moreover, functional groups enable the easy combination of biopolymers with different types of nanocarriers obtained through physical interactions or chemical conjugation. This is often carried out with stimuli-responsive chemical linkers that guarantee the detachment of biopolymer coatings in response to different triggers, enhancing the sensitivity or allowing the formulation of multi-responsive DDS [129,130,131,132]. Biopolymeric coatings are also good substrates for further functionalization, and several literature studies report their application in combination with different targeting molecules in actively targeted DDS [133,134,135,136]. 

## 4. Functionalization of MSNs with Biopolymers

Some relevant examples of DDS based on MSNs functionalized with natural biopolymers, categorized according to the chemical composition of the biopolymeric coating, are listed in Table 1 and discussed in the following sections. In particular, MSNs coated with protein-based biopolymers and natural polysaccharides will be discussed, describing their application in response to endogenous stimuli for the treatment of different diseases.

### 4.1. MSNs Coated with Polysaccharides

**Figure 2:**
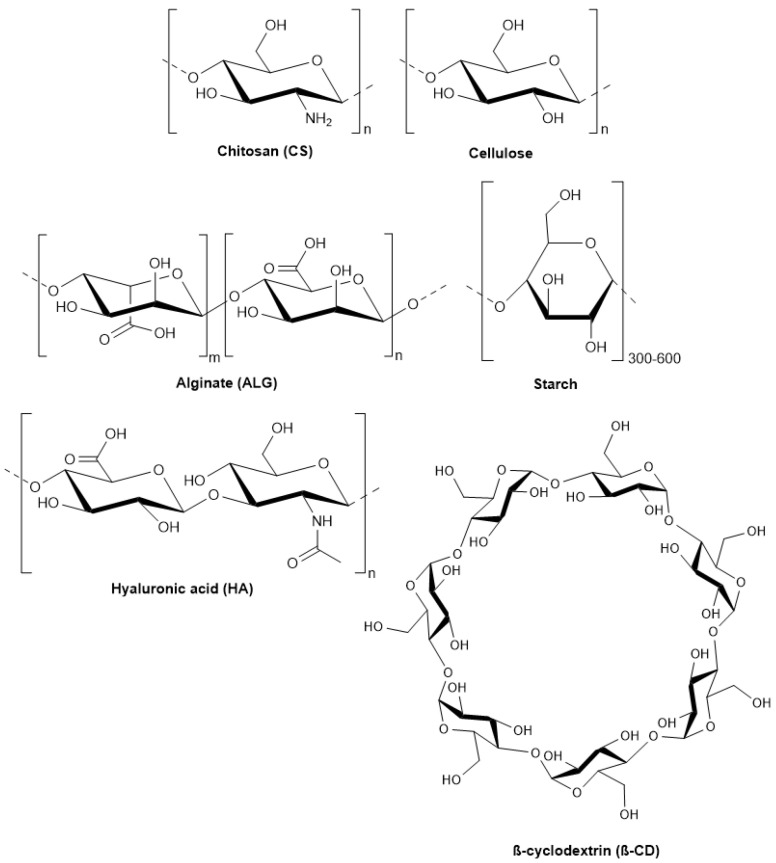
Chemical structure of natural polysaccharides.

Different types of natural polysaccharides derived from green and marine plants, animals, or microorganisms are applied as coating material in stimuli-responsive DDS. The class of polysaccharides includes linear polymeric carbohydrate structures formed by monosaccharides or disaccharides repetitive units linked through glycosidic bonds, as shown in Figure 2. 

#### 4.1.1. Chitosan

Chitosan is a linear polysaccharide composed by acetylated and deacetylated units (N-acetyl-D-glucosamine and D-glucosamine, respectively) randomly alternated and linked through β-1,4-glycosidic bonds (Figure 2). It is obtained by alkaline or enzymatic deacetylation of chitin, an abundant natural polymer found in the exoskeletons of crustaceans and insects and in fungal cell walls [165]. Depending on the source and production process, chitosan presents different structural features and the term actually indicates a family of polymers with different molecular weight and degree of deacetylation [166]. The degree of deacetylation, in particular, deeply affects the physical and biological properties of chitosan since it determines the amount of NH_2_ groups in the polymer chain, directly responsible for the solubility and charge of the compound [167]. Thanks to the presence of primary amines, chitosan exhibits a polycationic behavior which differentiates it from other natural polysaccharides and favors its interaction and uptake by negatively charged cell membranes [168]. Chitosan coatings are also advantageous for the intracellular distribution of drugs by promoting the endosomal escape of encapsulated therapeutic cargo due to the proton sponge action of the cationic biopolymer [139]. Moreover, the presence of NH_2_ groups together with OH groups ensures the high chemical reactivity of chitosan which can be easily modified with different functional molecules, such as stealth [169,170,171] or targeting [140,172,173] moieties.

As a natural material, chitosan is considered biocompatible, biodegradable, and non-toxic [174], but despite these promising features, the FDA has only approved it for wound dressing and dietary applications [175]. This may be explained by the huge variety of chitosan forms and derivatives which makes the systematic evaluation of its biocompatibility difficult [176]. Literature studies demonstrated the high cytocompatibility of chitosan-coated MSNs both in vitro [177,178] and in vivo [114,141], and their application as biocompatible smart nanocarriers has been widely reported in the literature. Exploiting the high chemical reactivity of both MSNs and chitosan chain, the biopolymer can be covalently grafted on the silica surface using different crosslinkers such as glycidoxypropyltrimethoxysilane [138,142,179], aminopropyltriethoxysilane [180,181], or by EDC/NHS chemistry [182,183]. In a different approach, the coating of MSNs can be achieved by physical absorption of chitosan on the NPs surface via electrostatic interactions between the positively charged polymer and the negative surface of MSNs [134,184]. Similarly, electrostatic interactions have been used to combine chitosan with other negative biopolymers such as hyaluronic acid [185], k-carrageenan [186], or alginate [187], through a layer-by-layer (LbL) self-assembly technique. This method allows composite coatings by the alternate deposition of layers of oppositely charged polymers around the MSNs surface to be obtained, finely regulating the thickness and composition of the coating, and subsequently controlling the release profiles [144].

Several studies report pH as the main endogenous trigger for cargo release from chitosan-coated MSNs [142,188,189]. The pH-responsiveness is provided by the variation in the protonation state of chitosan amino groups. Indeed, at high pH the –NH_2_ groups are deprotonated, and no electrostatic repulsions are present with chitosan chains compactly wrapped around the MSNs surface sealing the pores. In contrast, at pH < 6, the amino groups become protonated, leading to a swelling of the cationic polymer chains and allowing the release of the encapsulated payload [188]. The sensitivity to acid pH endows chitosan-coated nanocarriers with preferential release toward tumoral areas, which present an acidified environment compared to normal tissues [190]. Indeed, chitosan-coated MSNs has been widely investigated as pH-responsive DDS for potential cancer treatment and recent studies reported their application in the selective release of antitumoral molecules, such as doxorubicin [137,139], curcumin [138], and antimiR [139] (Table 1). The synergy between pH and redox stimuli has also been reported [140]. The combination of acidic pH and GSH guaranteed the in vitro and in vivo effective release of doxorubicin and tariquidar from manganese-doped MSNs coated with chitosan, acting both on the biopolymeric capping and on the doped silica network which was rapidly degraded under reducing conditions.

Enzymatic catalytic activity was indicated as another possible trigger for chitosan-coated smart DDS [191]. The release of doxorubicin was enhanced, incubating chitosan-coated hollow MSNs with colon enzymes [141]. Similarly, a fast release of Rhodamine B and doxorubicin from coated-MSNs was observed thanks to the combination of pH and lysozyme, an enzyme part of the innate immune system and overproduced by cancer cells. The synergy of the two stimuli led to rapid hydrolysis of the glycosidic bonds in chitosan chains, causing their decomposition into monomers and opening the MSNs pores [182].

**Figure 3:**
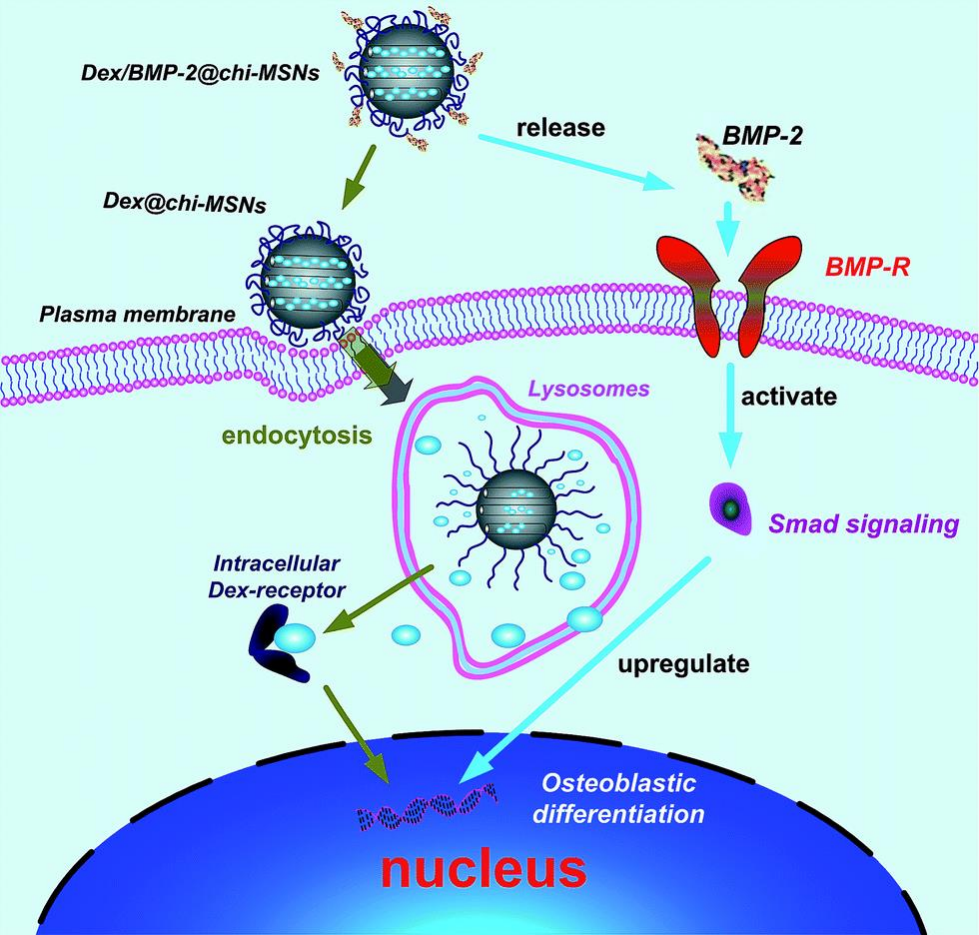
Schematic representation of the effect of chitosan-coated MSNs loaded with bone morphogenic protein-2 (BMP-2) and dexamethasone (Dex) in promoting osteoblasts differentiation. (Dex/BMP-2)@chi-MSNs are able to delivery BMP-2 extracellularly and Dex intracellularly, after the conformational change of chitosan coating triggered by lysosomal acidic pH. Reproduced with permission from ref. [142]. Copyright 2013, The Royal Society of Chemistry.

Besides cancer, chitosan-coated MSNs have been investigated for other biomedical applications, such as oral delivery of protein vaccines [192] or controlled release of antibiotic and silver ions in antibacterial applications [184]. A detailed study also analyzed the application of chitosan-coated MSNs in bone regeneration, showing enhancement in osteoblast differentiation in vitro and osteoinductive effect in vivo [142]. In this study, chitosan was exploited as gatekeeper for the pH-controlled release of dexamethasone, encapsulated in the MSNs pores, and as matrix for the immobilization and extracellular delivery of a bone morphogenic protein (BMP-2), as depicted in Figure 3. Moreover, the role of chitosan in supporting mineralization and osteoinductivity [193,194] was also accounted for, underlining the multiple features of the natural biopolymer.

#### 4.1.2. Alginate

Alginates are salts of alginic acid, a natural occurring polysaccharide obtained from cell walls of brown seaweeds [195]. Depending on the source, the structure of alginic acid may vary but it is essentially composed by a sequence of L-guluronic and D-mannuronic acid residues arranged in several proportions and geometries [196]. The alginic acid is extracted from marine algae by solubilization with dilute alkaline solution followed by treatment with mineral acids and it is subsequently converted into salts, among which sodium alginate is the most common [197]. The elimination of contaminants, such as heavy metals, endotoxin, and polyphenolic compounds, can be achieve through multistep extraction procedures which allow precise control of the composition and purity of the biopolymer [198].

The biocompatibility, low toxicity and low immunogenicity of sodium alginate is recognized and it is already applied as excipient in several commercialized drugs [199] and widely investigated in the field of drug delivery [200]. As shown in Figure 2, alginate is characterized by the presence of carboxylic groups in its structure which are responsible for its high solubility in water at neutral and alkaline conditions and its sensitiveness to pH stimuli [201]. In reverse to what was observed for chitosan, alginate matrix expands to a pH higher than 4.4 with a maximum at physiological pH due to the occurrence of electrostatic repulsion between ionized –COO^−^ groups. On the other hand, in strongly acidic conditions, the carboxylic groups are non-ionized and alginate precipitates as a water insoluble structure [202]. This behavior confers to the biopolymer a release ability in response to pH increase, which can be exploited for controlled delivery in oral administration. Alginate-coated MSNs were studied as smart nanocarriers for oral delivery of poorly soluble drugs. The alginate coating was proven to resist to acid buffer, mimicking the condition of gastric fluids, preventing the premature release of drug molecules [203]. A sustained release was instead observed at higher pH, corresponding to intestinal fluids [203,204].

The coating with alginate is generally achieved by electrostatic interactions between the anionic biopolymers and the MSNs surface, made positive by appropriate functionalization [205]. Moreover, alginate is used in combination with positive polymers to create composite coatings by the already mentioned LbL self-assembly technique [206,207,208]. For instance, two recent studies used alginate in combination with chitosan for the formulation of pH-responsive polyelectrolyte-coated MSNs in the treatment of cancer [144] and neurodegenerative diseases [187]. The covalent bonding of sodium alginate to MSNs surface via EDC/NHS chemistry and disulfide linkers was also reported [143]. In this case, alginate-coated MSNs showed responsiveness to pH and redox stimuli for the controlled release of doxorubicin in antitumor application. The alginate coating was proven to limit the premature cargo release, while it was efficiently removed through the cleavage of disulfide bonds after the application of acid pH and GSH molecules, mimicking the conditions of tumor microenvironment [209]. Moreover, the negative charge provided by alginate coating was reported to prevent protein absorption and enhance blood circulation time of the coated DDS.

#### 4.1.3. Hyaluronic Acid

Hyaluronic acid is a linear biopolymer made by disaccharide repeated units of d-glucuronic acid and d-*N*-acetylglucosamine linked through β-glycosidic bonds (Figure 2). It belongs to the class of glycosaminoglycans, negative unbranched heteropolysaccharides naturally produced by higher organisms [210]. Hyaluronic acid is a major constituent of the extracellular matrix of connective, epithelial, and neural tissues [211], and it can also be found in the extracellular capsule of some microorganisms, such as wild strains of *Streptococcus* [212]. Actually, the microbial source has emerged as a valuable option for the industrial production of hyaluronic acid, replacing traditional animal sources, such as rooster combs or bovine vitreous humors [213]. In this method, hyaluronic acid is produced by bacteria through the fermentation of lignocellulose feedstocks and then recovered from extracellular broth by precipitation, followed by purification and refinement steps [214]. Compared to extraction from animal sources, microbial fermentation guarantees a reduction of costs and environmental impact as well as higher yield and product quality with a simple process flow. In addition, animal-derived hyaluronic acid has high immunogenic and allergic potential, while the use of *Streptococci* or non-pathogenic recombinant strains can minimize these safety concerns and provide more suitable products for biomedical and pharmaceutical applications [212]. 

Hyaluronic acid has been investigated in the field of drug delivery thanks to its high biocompatibility and ability to extend blood circulation time [215,216,217]. Moreover, it shows ability for controlled release and cell-targeting, which make it particularly interesting for the design of specific smart DDS in cancer treatment. Indeed, hyaluronic acid-binding receptor CD44 is overexpressed by various cancer cells such as ovarian, breast, colon and squamous carcinoma cancer cells [218], and has been shown to increase the mediated uptake of several types of hyaluronic acid-modified NPs, including MSNs [219,220,221]. For instance, recent in vivo studies employed hyaluronic acid-coated MSNs for the controlled release of 5-fluorouracil [146] and a combination of MTH1 inhibitor and siRNA [222], reporting superior effect when compared to uncoated MSNs in the treatment of colon cancer and oral squamous carcinoma, respectively. The selective in vitro and in vivo uptake of hyaluronic acid-coated MSNs by CD44-positive breast cancer cells was reported [145]. In this study, the biopolymer-coated particles were responsive to the acidic pH of tumor microenvironment, showing a significantly higher release of doxorubicin at pH 5.5 compared to physiological pH. 

However, the most reported trigger for hyaluronic acid-coated MSNs is the enzymatic stimulus as highlighted in Table 1. Hyaluronic acid molecules, characterized by high molecular weight, are proven to efficiently block the MSNs pores, minimizing the premature release of payloads in physiological conditions [146,222]. In tumoral areas, the biopolymer coating can be efficiently removed by the hydrolysis catalyzed in the first extent by hyaluronidase-2 (Hyal-2), present on cancer cells membranes, and then by hyaluronidase-1 (Hyal-1) after mediated endocytosis [223]. Due to the significantly high concentration of hyaluronidase in senescent aortic plaques, hyaluronic acid-coated MSNs have also been proposed for the treatment of atherosclerosis and the enzyme-responsive release of simvastatin [147] and rosuvastatin [148] in senescent foamy macrophages was recently reported. In both studies hyaluronic acid-coated MSNs were able to efficiently inhibit foamy macrophages proliferation and arrest plaque progression. Moreover, the active targeting of hyaluronic acid coating toward CD44 receptor of inflammatory macrophages was also demonstrated [147]. Similarly, the overproduction of hyaluronidase by bacteria in infection sites was exploited for the development of responsive DDS for the treatment of resistant bacterial infections [149]. Hyaluronic acid-coated MSNs allowed the controlled release of cinnamaldehyde bactericidal compound in mice infected with methicillin-resistant *Staphylococcus aureus* subcutaneous abscesses without harming the surrounding normal tissues.

**Figure 4:**
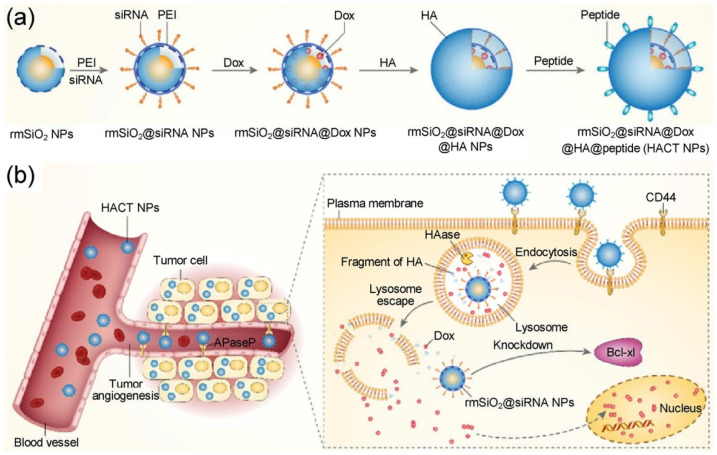
Schematic representation of: (**a**) construction of rattle-MSNs (rmSiO_2_ NPs) coated with PEI and hyaluronic acid (HA) through “layer-by-layer assembly” strategy, loaded with doxorubicin and siRNA and functionalized with cell-penetrating peptide; (**b**) mechanism of selective controlled release guaranteed by the cascade of targeting moieties (cell-penetrating peptide and HA) and triggered by hyal-1. Reproduced with permission from ref. [229]. Copyright 2016, Springer Nature.

More complex DDS based on MSNs coated with hyaluronic acid in combination with synthetic polymers were also studied. For instance, Janus MSNs coated with hyaluronic acid and 2,3-dimethylmaleic anhydride (DMMA) were formulated as dual-responsive smart DDS [224]. The presence of hyaluronic acid successfully improved the biocompatibility of the nanosystem, and the two polymers synergistically contributed to the controlled release of doxorubicin in response to acidic pH (for DMMA) and hyal-1 (for hyaluronic acid). Moreover, the use of hyaluronic acid together with polyethylenimine (PEI) has also been reported [225,226,227,228]. An in vivo study employed rattle-MSNs coated with a multilayer of PEI and hyaluronic acid for the co-delivery of siRNA and doxorubicin to breast cancer cells [229]. As depicted in Figure 4, the hyaluronic acid coating provided targeting towards the CD44 tumoral marker and was exploited for further conjugation of a cell-penetrating peptide, responsible for the preferential accumulation in tumoral vasculature. As well, the hyaluronic acid coatings guaranteed the controlled release triggered by Hyal-1 in lysosomes, while the presence of PEI favored the lysosomal escape of drugs. 

#### 4.1.4. Cellulose and Starch-Derived Dextrin and β-Cyclodextrin

Cellulose, as a main component of primary cell wall of plants and some types of algae and fungi, is the most abundant natural polysaccharide. It is composed by glucose monomers linked through β-1,4-glycosidic bonds to form linear polymer chains (Figure 2). Cellulose can be extracted from various plant materials, including agricultural residues, which are particularly attractive as renewable and low-cost sources by different methods. These include alkali treatment, enzymatic-assisted extraction or mechanical treatments based on the use of ultrasounds, microwaves, or high pressure [230]. Cellulose with a high degree of purity is also produced by bacterial digestion of natural and synthetic sugar feedstocks [231].

As an eco-friendly, renewable, and non-toxic material, cellulose finds its application in many technological fields. Thanks to its biocompatibility and high mechanical strength, cellulose is extensively investigated for tissue engineering applications [232,233] but its use as a stimuli-responsive material in smart DDS has also been reported [122,234]. Cellulose can be biodegraded into small glucose units by lysosomal glycosidase enzymes, and this feature was exploited to achieve controlled release of doxorubicin from cellulose-coated MSNs [150]. In this study, cellulose was conjugated to MSNs surface through esterification using the -OH groups present in the biopolymer chains. In vitro tests demonstrated the ability of cellulose coating in preventing the premature drug release under physiological conditions and the delivery in response to enzyme and pH triggers, capable of hydrolyzing the biopolymer chains and ester bonds, respectively. 

**Figure 5:**
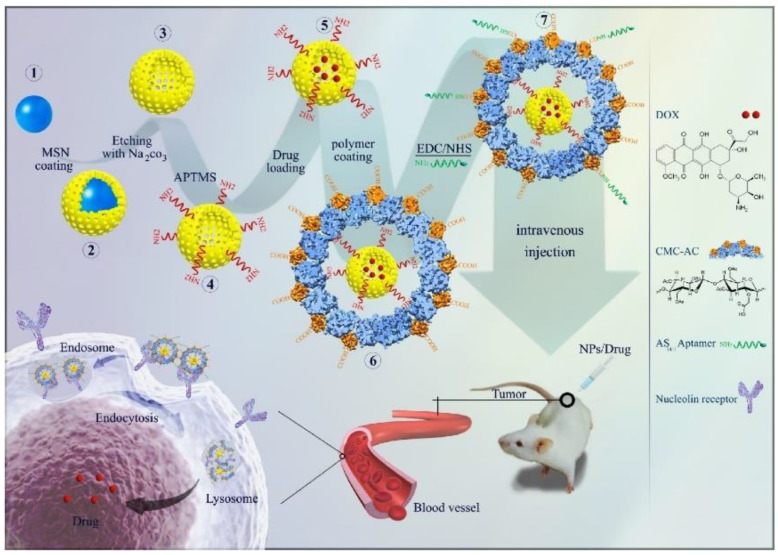
Schematic representation of the functionalizing process (**top**) and targeting and drug release mechanism (**bottom**) of hollow MSNs coated with acetylated carboxymethylcellulose (Ac-CMC) decorated with aptamer. Reproduced with permission from ref. [151]. Copyright 2018 Elsevier Ltd.

More soluble derivatives were also investigated and, for instance, carboxymethylcellulose (CMC) was applied to coat MSNs surface, ensuring an enhanced uptake and efficient delivery of curcumin in breast cancer cells [235]. Similarly, hollow MSNs coated with acetylated CMC showed a controlled release of doxorubicin in response to acid pH stimulus [151], as schematized in Figure 5. The carboxylic groups of CMC were also exploited to conjugate aptamer molecules specific for nucleolin receptor overexpressed by some tumor cells, obtaining preferential accumulation and enhanced cytotoxic effect both in vitro and in vivo.

Starch is another glucose-based biopolymer synthesized by plants. Differently from cellulose, the starch glucose monomers are linked by α-glycosidic bonds (Figure 2) to form linear or branched chains named amylose and amylopectin, respectively [236]. It is generally extracted from plant seeds, roots, and tubers through steps of physical separation that progressively eliminate cellulose, proteins, and other impurities [237]. Limitations, mainly related to the heterogeneity and agglomeration tendency of natural starch, affect the performance of the biopolymer in drug delivery applications. Thus, several chemical and biochemical modifications to obtain starch derivatives with improved solubility and reactivity have been implemented [236]. 

For instance, dextrin, characterized by lower molecular weight and higher water solubility due to the destruction of starch crystalline structure, can be produced by hydrolysis processes. Recent studies applied oxidized dextrin crosslinked with cystamine as MSNs coating agents for the controlled release of doxorubicin in response to pH and redox stimuli [238,239]. 

Another starch derivative widely used in biomedical applications is β-cyclodextrin. It is a monocyclic structure composed by seven glucose monomers linked through α-1,4-glycosidic bonds (Figure 2) generated through enzymatic conversion of starch by cyclodextrin glycosyl transferases (CGTases) and α-amylases [240]. Its use in drug delivery applications is supported by facile chemical modification, good biocompatibility, and absence of toxicity. Moreover, the particular structure of cyclodextrins endows them with an amphiphilic nature, characterized by a hydrophilic exterior and a hydrophobic cavity. This can host different hydrophobic molecules to form inclusion complexes based on host-guest interactions, which can be modulated by the application of different internal and external stimuli, thus regulating the cavity opening [241]. Considering this feature, inclusion complexes of β-cyclodextrin with different guest molecules have been intensively investigated as gatekeepers for the formulation of smart DDS [242,243]. 

**Figure 6:**
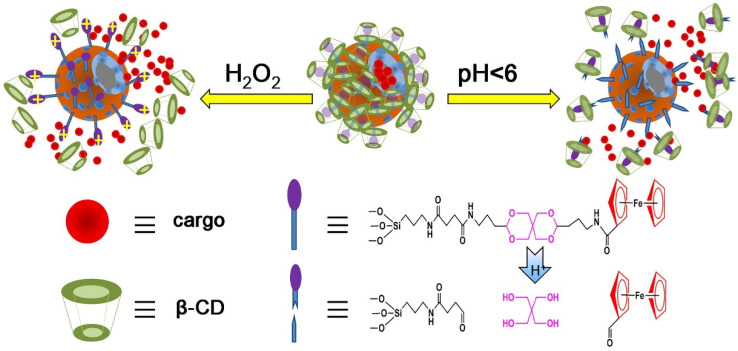
Schematic representation of the mechanism for controlled release from MSNs coated with dual-responsive β-cyclodextrin nanovalves. The biopolymeric coating responds to H_2_O_2_, acting on β-cyclodextrin/ferrocenyl complexes (**left**), and to pH acting on ferrocenyl acetal linker (**right**). Reproduced with permission from ref. [244]. Copyright 2016 Elsevier Inc.

Recently, mesoporous silica nanorods grafted with phenylboronic acid pinacol ester (PBAP) complexed with β-cyclodextrin were investigated for the controlled delivery of doxorubicin [152]. The β-cyclodextrin molecules acted as effective gatekeepers avoiding the premature drug release in physiological conditions while they were open after the exposure at tumoral acidic environment by the weakening of host-guest hydrophobic interactions with PBAP. This allowed the initial release of doxorubicin, which was further increased by the redox degradation of H_2_O_2_-sensitive PBAP, providing in vitro and in vivo effective responsive release. Inclusion complexes of β-cyclodextrin and ferrocenyl moieties were also evaluated for the responsive release of molecules with different size (i.e., Rhodamine 6G, doxorubicin and 5- fluorouracil) from coated MSNs [244]. As schematized in Figure 6, the dissociation of the complex was triggered by the oxidation of ferrocenyl by H_2_O_2_ species, which produced strong electrostatic repulsions. The drug release was also supported by acidic pH acting on acetal linkers used to connect the ferrocenyl moieties to silica surface. 

In another research, hollow MSNs functionalized with β-cyclodextrin were applied as smart DDS for the transdermal delivery of an anti-pigmentation agent to treat skin conditions [153]. In this study, host-guest interactions were exploited to anchor a cell-penetrating peptide, used to overcome the skin barrier. The β-cyclodextrin were then conjugated to silica surface via boronic acid-catechol ester bonds responsive to ROS trigger. Considering the high level of oxidative stress in UV-exposed skin, this was a very suitable stimulus, and the developed DDS showed good penetration and delivery abilities both with in vitro and in vivo models, also demonstrating a high colloidal stability and biocompatibility.

### 4.2. MSNs Coated with Protein-Based Biopolymers

**Figure 7:**
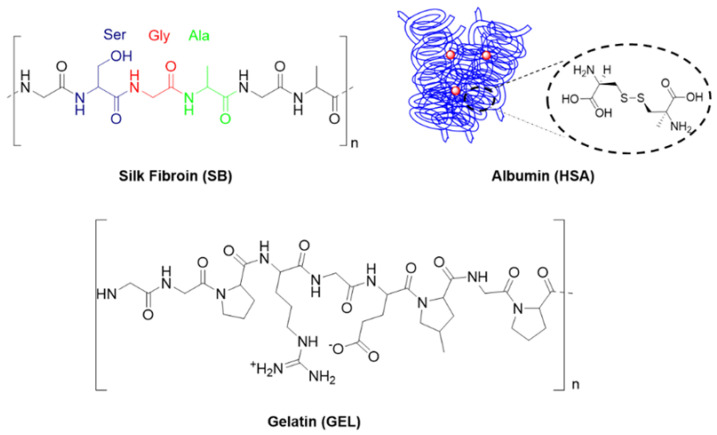
Chemical structure of proteinaceous biopolymers.

Animal originated proteins such as silk fibroin, collagen, gelatin, and albumin are widely used for drug delivery systems. As depicted in Figure 7, these kinds of proteins are high molecular weight compounds.

#### 4.2.1. Silk Fibroin 

Silk Fibroin is a natural macromolecular protein derived primarily from silkworm cocoons (*Bombyx mori*), but also from spiders (*Nephila clavipes*). Its structure consists of a block copolymer that contains 26 KDa light and 390 KDa heavy chain joined by a disulfide bridge [245,246]. Specifically, it is a β-sheet structure self-assembled of repetitive chains of amino acids Gly–Ala–Gly–Ala–Gly–Ser (Figure 7) [247].

Silk Fibroin can be purified from native silkworm cocoons by removing proteins and debris that have the potential to cause inflammatory responses. The more common procedures of purification and fabrication include chemical solvents, energy-intensive equipment, and large quantities of water. A typical methodology used to reverse engineer silk fibroin into an aqueous solution and finally yielding the final manageable material format. 

It is important to note the degumming process used to purify SF fibers [248]. This process the cleavage of the peptide bond of sericin, a glue-like proteinaceous substance that covers the fiber, either by hydrolytic or enzymatic methods. Typically, alkaline solution containing soap is employed for the hydrolysis of sericin, but it can also be carried out under neutral or acidic conditions to give four fractions, each having different properties. This enhances the innate properties of SF such as biocompatibility, tunable degradability, low toxicity, and mechanical properties. 

Its favorable natural characteristics have provoked that SF had been extensively explored as a potential biomaterial in biotechnological and biomedical fields. In fact, chemical and physical properties of SF make it a suitable capping and active targeting agent for improving the application of MSNs as DDS.

In a study reported by Khosravian and coworkers [154], the surface of MSNs was coated with SF for encapsulating antitumor drug TG in their mesopores. The in vitro results showed an increase in loading capacity and biocompatibility and a successful delivery of the hydrophobic anticancer drug TG when the nanosystem is under acidic condition at tumoral ambient. This situation produces the degradation of silk; thus, SF could be a promising capping agent with pH-sensitive targeting release as well an active targeting agent.

#### 4.2.2. Gelatin 

Gelatin, a natural proteinaceous biopolymer, is obtained from the partial hydrolysis of collagen that is derived from the skin or bones of different animals [249]. It is possible to obtain gelatin with a different electrical nature depending on the processing method used from a collagen source: the hydrolysis of amide groups of collagen in the alkaline process makes the gelatin negatively charged, due to high density of carboxyl groups present in its structure, and a reduced isoelectric point (IEP) is obtained. In contrast, the less invasive acid process through amide groups of collagen highly modifies their electrostatic nature and remains a similar IEP for gelatin [250,251].

There are two types of gelatin: Type A and Type B. Type A gelatin is derived from acid hydrolysis of collagen and it has 18.5% nitrogen, whereas Type B is derived from alkaline hydrolysis containing 18% nitrogen and no amide groups.

**Figure 8:**
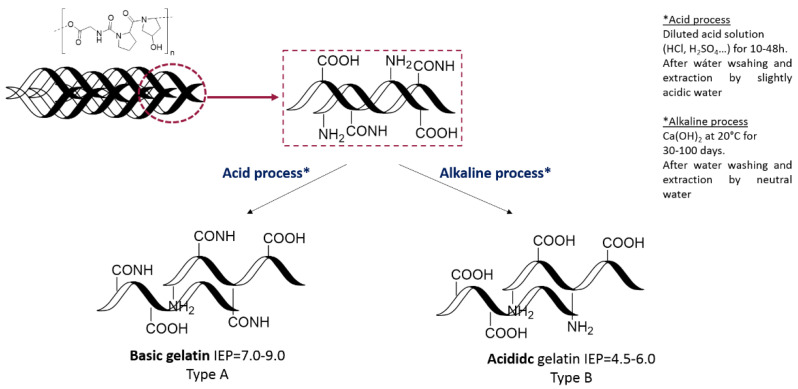
Collagen processing method for yielding gelatin samples with different IEP values.

As summarized in Figure 8, the basic composition of gelatin contains great amounts of amine, carboxyl, and amide functional groups and hence, this protein could be negatively or positively charged upon pH change. This functional diversity allows it to be modified by using nanoparticles and biomolecules. In addition, gelatin is an extracellular matrix protein which allows it to be applied in wound dressings, drug delivery, and gene transfection [252,253,254]. It presents desirable features, such as natural origin, low-cost, low-toxicity, biodegradability, and non-immunogenicity, although it shows poor stability in aqueous solutions [255].

One of the most effective methods to increase their thermal and mechanical properties and lower their aqueous degradation is to use chemical crosslinking agents such as glutaraldehyde (GA), among others [249]. This strategy consists of bridging free amine groups from the gelatin with carbonyl groups from aldehyde and modifying the cleavage sites of gelatin molecules in order to perform a higher reticulation which increases particle size. This network allows its hydration potential under physiological conditions, as well as lowering its degradation in in vivo environment. Moreover, depending on the crosslinking density of the gelatin, it is possible to alter and regulate control release functions as a coating-carrier [81].

In a study reported by Wang and coworkers, a pH-responsive system for intracellular anticancer drug–controlled release based on gelatin capped nanoparticles was developed. The gelatin layer of coating was performed grafting the gelatin onto the MSN via gelation and subsequent glutaraldehyde cross-linking [127]. Then, it was demonstrated that the anticancer drug doxorubicin (DOX) was encapsulated into mesopores at physiological ambient, but in slightly acidic conditions, the pores were opened for the rising of electrostatic repulsive force between the gelatin and MSNs, and it was allowed the diffusion of DOX molecules in cancer cells. Thus, this study demonstrated the gatekeeper function of gelatin for designing a pH responsive intracellular drug delivery nanocarrier.

**Figure 9:**
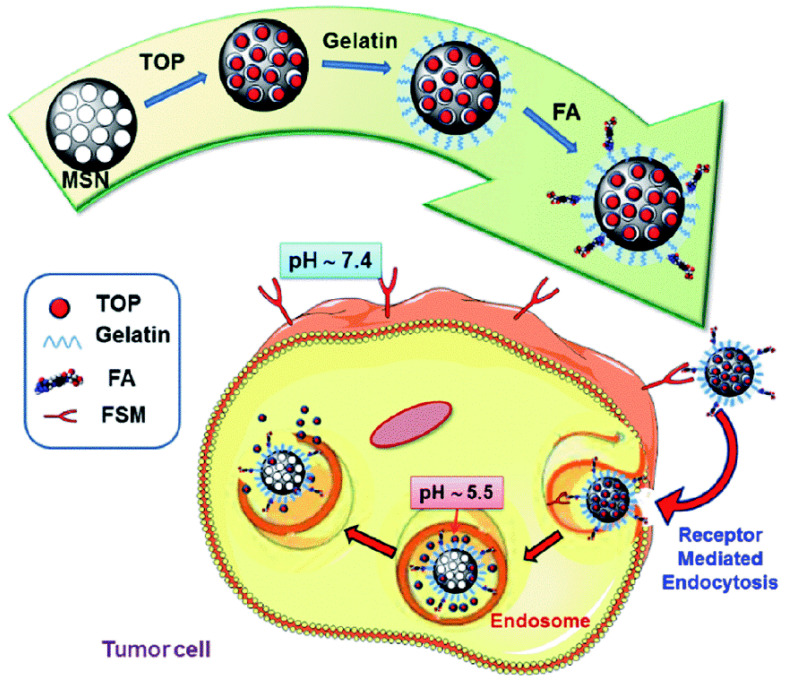
Schematic illustration of the tumor-targeted TOP nanosystem consisting of MSNs encapsulating TOP, coated with a gelatin shell and decorated with folic acid (FA) as targeting ligand. FSM stand for surface markers with affinity toward FA, which are usually overexpressed in the membrane of certain tumor cells. Reproduced with permission from ref. [155]. Copyright 2011, The Royal Society of Chemistry.

In a posteriori study by Vallet-Regí et al., authors provided a targeted pH-sensitive nanosystem based on coated-MSNs with a gelatin shell in order to selectively deliver TOP to tumor cells [155]. In this case, the gelatin coating process was similar to previous studies, but with further improvement since the gelatin multifunctional shell was designed to facilitate the grafting of a targeting agent, and also to close the mesopores, preventing the premature TOP release. The results indicated that gelatin was capable of acting as a pH-sensitive layer because it was triggered at the acidic conditions (endo/lysosomes; pH ≤ 5.5), providing an efficient release of active form of TOP in tumoral cells (Figure 9).

**Figure 10:**
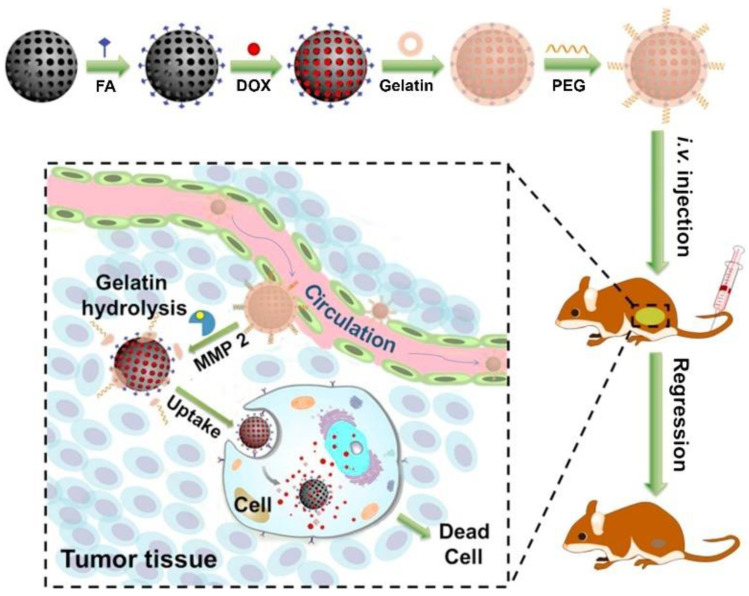
Schematic illustration for the formulation of multifunctional drug-loaded PGMSN and MMP 2-triggered targeting and drug delivery strategy of PGFMSN. Reproduced with permission from ref. [103]. Copyright 2015, Elsevier Ltd.

It is well documented that MMP2 is also extremely efficient at hydrolyzing gelatin [256]. Therefore, using gelatin as MMP2-sensitive linker has great potential to construct PEGylated and tumor-targeted drug nanocarriers for in vivo applications [103]. Zou et al. developed a smart and multifunctional mesoporous silica nanocarrier (PGFMSN) loaded with an anticancer drug DOX and rational packaging designed for tumor targeting and enzymatic responsive stimuli [103]. It was constructed in sequential steps (Figure 10): (i) firstly, they used folic acid (FA) as a target agent in order to improve cellular uptake. (ii) Then, via temperature-induced gelation and crosslinking agent (glutaraldehyde, GA), the gelatin was grafted and introduced onto FA-MSN. Thus, at the same time, the gelatin layer caps the mesopores and protects the targeting agent. (iii) Finally, gelatin-coated MSNs were further decorated to prolong blood-circulation lifetime via PEGylation.

In vitro results demonstrated that this system can specifically target cancer cells responding to the up-regulated extracellular MMP 2, since this enzyme hydrolyzes the gelatin layer and deshields the PEG layer. As a result, the function of FA is activated for enhancing the selective uptake by tumor cells mediated endocytosis, thanks to folate receptors. Additionally, the enzymatic triggered response of the nanocarriers is responsible of the successful release of DOX in the tumoral microenvironment due to the degradation of gelatin, thus compared with gatekeeper-free systems, PGMNS exhibited a better therapeutic efficacy.

Another example of novel intelligent DDS was designed by Zhang and Xu using a MEND-strategy (multifunctional envelope-type nanodevice). The novelty of this study is the development of a new device capable of responding to two different enzymes for triggering the cargo on-demand [156].

**Figure 11:**
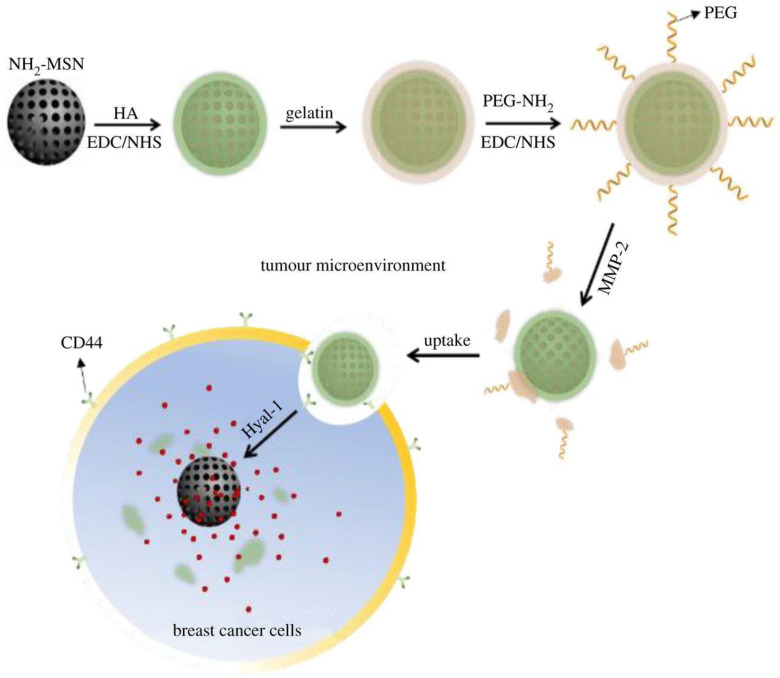
Schematic illustration of the formation of multifunctional drug-loaded MHGP and bienzyme-responsive tumor targeting and controlled release. Reproduced with permission from ref. [156] under the Creative Commons attribution (CC-BY) License.

MMP-2 overexpressed outside of the tumor cells (Figure 11) catalyzes the degradation of the gelatin layer exposing the hyaluronic acid coating, which acts both as a targeting and capping agent. Once inside the tumor microenvironment, drug release is then triggered by Hyal-1. This system presents successful bienzyme-responsive targeted drug delivery for clinical cancer therapy and cancer pharmaceutical development.

#### 4.2.3. Albumin

Albumin, also named human serum albumin (HSA), is the most abundant protein in the human blood plasma (35–50 g/L human serum). It contains 585 aa, 17 disulfide bridges, and has a molar mass of 66.5 KDa. In humans, albumin synthesis takes place in the liver and it is negatively charged.

Therefore, albumin is an endogenous human substance whose main characteristic is its high biocompatibility [257,258]. This property has been widely exploited by many researchers in recent years focusing on the ability of albumin to facilitate the internalization of the drug, increase its solubility and obtain a sustained drug release profile [158,259].

**Figure 12:**
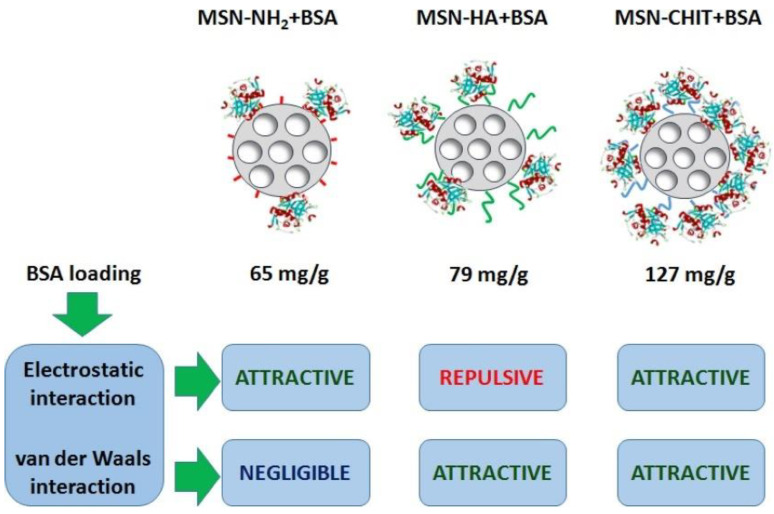
Schematic illustration of the interactions responsible of the formation of the protein corona on functionalized MSNs. Reproduced with permission from ref. [265]. Copyright 2018, Elsevier B.V.

Several studies have shown that HSA association with surface modified MSNs can be a beneficial approach to enhance nanoparticle internalization. It was observed that the extent of HSA adsorption on the MSN surface varied depending on the surface modification of MSNs [260,261,262]. The principle of albumin coating of mesoporous silica nanoparticles is based on electrostatic adsorption. At pH 7.4, an albumin molecule has more than 200 negative charges, whereas the MSNs with amino groups are positively charged. Due to electrostatic interactions, the pores of MSNs can be sealed by albumin [263]. Karamana et al. used different surface-modified MSN-PEI/PEG to study the interaction mechanism of HSA with MSN analogs. They found that the association with HSA finely regulates the cellular internalization of MSNs and for that reason it represents a beneficial approach for surface modified MSN for stem cell-based tissue engineering research due to the provided, MSN colloidal stability, improved stem cell viability, stem cell internalization, and lack of adverse effects on the differentiation of stem cells [264]. Another interesting report used MSNs coated with bovine serum albumin (BSA) together with other kind of biopolymers that result in a different MSN surface charge to analyze how surface functionalization influences the adsorption mechanism of albumin in order to obtain protein corona surface. They remark the overall importance on the biological systems, of the electrostatic and non-electrostatic charge interactions likely cooperate to get the highest BSA loading on functionalized-MSNs (Figure 12) [265].

Another advantage of albumin-coated MSNs is the potential to overcome mononuclear phagocytic system (MPS), a key participant in determining the half-life of nanoparticles in the bloodstream and influencing active targeting. In this case, one study is focused on avoiding the MPS using this coating strategy with MSNs in a mouse model of endometrioses [266].

Because of all of the above-mentioned characteristics, albumin coating has been combined with other molecules or synthetic polymers in order to obtain effective drug delivery systems with MSNs as nanocarriers [158,259,267]. Most of them have been studied for the transport of anticancer drugs with poor pharmacokinetic profile and metabolic stability such as DOX, PTX, and DTX, among others [263,268].

Two representative examples could be highlighted. On the one hand, Dong and co-workers designed folate targeting and bovine serum albumin gated mesoporous silica nanoparticles as a redox-responsive carrier for epirubicin [157]. In this study, they combined BSA with MSNs via a simple amidation process and folic acid targeting. Then, they applied reduction-responsiveness by glutathione for triggering payload on-demand in cancer cells, exploiting the BSA natural disulfide bonds and abundant amino and carboxyl groups. In vitro results showed that high concentration of GSH provokes that the disulfide linkages in BSA can be bio-cleaved, thus allowing the delivery of EPI entrapped in the pores. In addition, this nanosystem also exhibited a higher cellular uptake by HepG2 cells than PC-12 cells via the folate receptor-mediated endocytosis. 

On the other hand, Tehrani et al. published a report where a pH sensitive bilayer coating was synthesized for controlled delivery of gemcitabine to cancer cells [158]. The bilayer was formed by Poly(acrylic acid-*co*-itaconic acid) as an inner shell and HSA as an outer shell around the mesoporous silica. They proposed that the acidic pH responsive drug release of copolymer could be due to electrostatic interaction between them and mesoporous silica surface. At endosomal pH, the configuration of the copolymer shell changed to a collapsed state and repulsive electrostatic interaction between shell and MCM-41 also occurred. Thus, both events facilitated releasing from the pores and turn it into a potential smart candidate for cancer therapy.

### 4.3. MSNs Coated with Other Biopolymers

**Figure 13:**
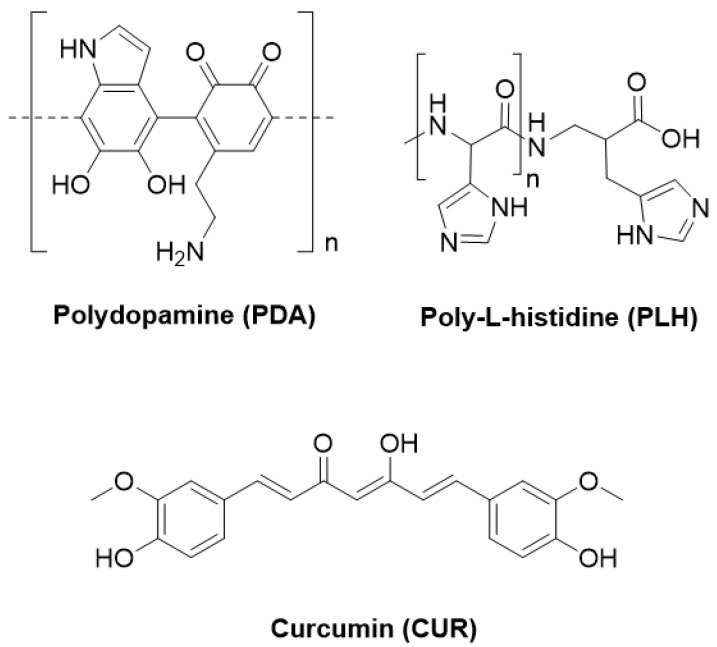
Chemical structure of Polydopamine, Poly-L-histidine, and curcumin.

Other polymers applied as coating agents in drug delivery applications are polydopamine (PDA), poly-(L-histidine) (PLH), and poly(curcumin). They are obtained through the polymerization of amino acids or secondary metabolites, and can therefore be assimilated to protein-based biopolymers, with which they share some structural features as shown in Figure 13.

#### 4.3.1. Polydopamine

**Figure 14:**
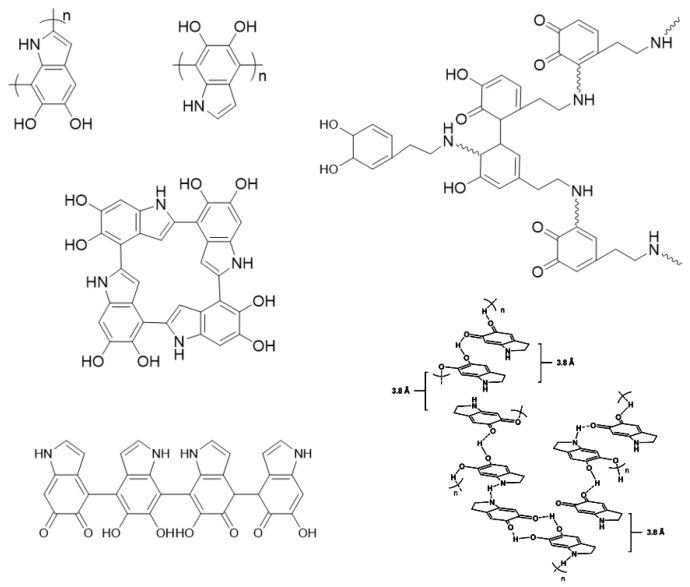
Schematic illustration of the other structures found in the literature for the PDA.

The polydopamine (PDA) is derived from the mussel adhesive protein-inspired precursor dopamine [269], and possesses many properties, such as a simple preparation process, good biocompatibility, strong adhesive property, easy functionalization, outstanding photothermal conversion efficiency, and strong quenching effect [270]. Autoxidation polymerization of dopamine allows for the creation of PDA coating layer on nanoparticles with different surface chemistry, enhancing the cellular uptake, cell affinity, and biocompatibility of coated nanoparticles. Moreover, PDA layers have been shown to increase the hydrophilicity of different organic and inorganic materials through multiple hydration mechanisms, mimicking real tissue conditions and finding their application in different biomedical fields, such as drug delivery, cancer therapy and regenerative medicine [271]. The complete elucidation of the structure of PDA is difficult due to the variety of reactions that can take place simultaneously, such as Michael additions, formation of Schiff bases, or radical reactions, among others [272]. For this reason, the structure is not identified in detail, but the groups that contains are well-known, such as quinones, catechol and amines. Figure 14 shows all the structures found in the literature [272,273,274,275].

The polymerization of catechol is carried out by the autoxidation of the catechol groups in a basic medium in the presence of air. In this polymerization, ammonia acts as a nucleophile on the o-quinones formed, accelerating the oxidation between catechol rings and therefore the crosslinking between them, giving rise to PDA [272].

Among the polymers used for coating nanocarriers, PDA has attracted increasingly considerable attention because it presents very interesting properties and a simple and versatile approach to functionalize material surfaces for obtaining a variety of multifunctional nanomaterials [115]. It is capable of being deposited on almost any type of surface, allows the anchoring of biomolecules, and shows high biocompatibility [269]. The presence of moiety catechol in its structure is the main reason for its efficacy in cargo released, since catechol groups also help the PDA framework strongly interact with aromatic drug molecules through π-π stacking and hydrogen bonds. Moreover, quinone moieties in polydopamine structure allow the easy immobilization of different biomacromolecules containing amino or thiol groups, such as peptides and protein and growth factors [271]. As a result, PDA had been extensively investigated to develop controlled-release formulations of anticancer drugs [161,276,277].

**Figure 15:**
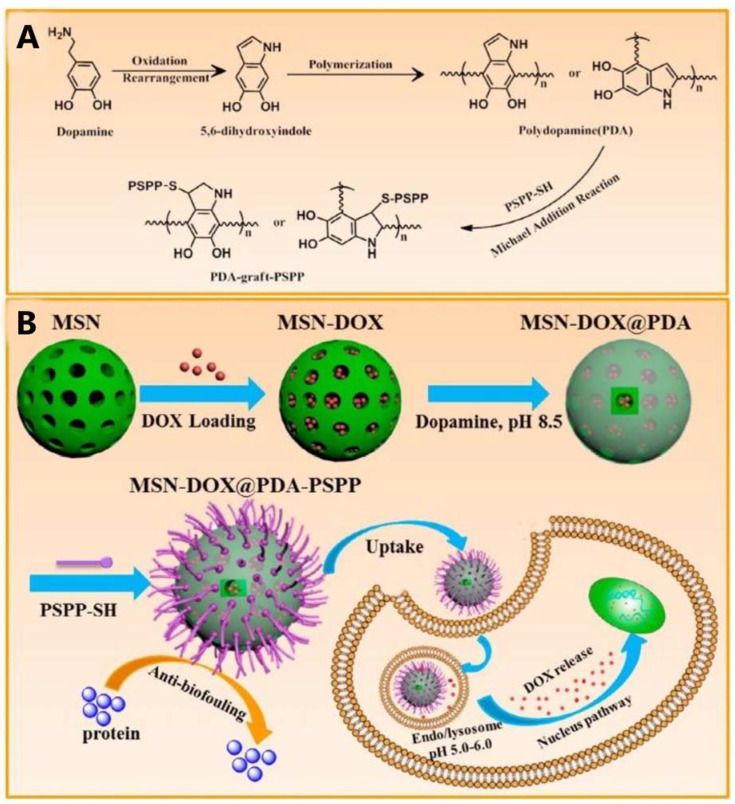
(**A**) Oxidative polymerization of dopamine and conjugation pf PSPP-SH with polydopamine (PDA) through Michael addition reaction; (**B**) Schematic illustration for the formation process of mesoporous silica nanoparticles-doxorubicin@PDA-PSPP (MSN-DOX@PDA-PSPP), the cellular uptake and drug release process of MSN-DOX@PDA-PSPP. Reproduced with permission from ref. [159] under Creative Commons Attribution (CC-BY) license.

In a study reported by Ji et al. a drug nanocarrier was engineered which possess high anti-cancer drug loading capability (DOX drug employed) and a pH-sensitive drug release performance due to the PDA coating [159]. The authors also decorated the outside surface of MSNs with a zwitterionic polymer (poly(3-(3-methacrylamidopropyl-(dimethyl)-ammonio)propane-1-sulfonate), (PSPP-SH)) that provides good anti-protein adsorption properties due to excellent stability in the complex physiological environments. This sophisticated design allowed to observe outstanding in vitro release of DOX molecules into the cell nuclei whose behavior was pH-dependent (Figure 15). 

Dual pH and ultrasound stimuli-responsive PDA-coated MSNs were prepared by Li and coworkers for cancer therapy. In this study, the coating of PDA over the MSN surface was obtained through oxidative self-polymerization under the alkaline condition. This nanocarrier showed accelerated drug delivery of doxorubicin under acidic conditions due to the degradation of PDA at low pH (pH 5.0) [278]. In fact, another study that used a similar coating strategy to yield hybrid MSN@PDA also expanded the feasibility of this platform in terms of exploring its ability to intracellular delivery of both a hydrophilic and hydrophobic (fingolimod, FTY720) anti-cancer drug, as well as investigate the effect of particle shape on intracellular release [279]. In a very recent report, it has been synthesized a pH-responsive glucosamine anchored polydopamine coated MSN for delivery of Anderson-type polyoxomolybdate in breast cancer [160].

There are multiple examples in the literature that not only outstand the acid pH-stimuli responsive role of PDA as gatekeeper for cargo release in MSNs, but also the synergistic effect achieved by its combination with other types of entities/moieties, such as other polymers, aptamers, or even metal complexes [280,281,282,283].

#### 4.3.2. Poly(L-histidine)

Poly(L-histidine) is an unsaturated nitrogen rich polymer that contain an imidazole group responsible for its amphoteric property through protonation/deprotonation [284,285]. Moreover, this biopolymer presents a high pH-response because undergoing chemical changes in response to environmental pH changes such as protonation, charge reversal, and bond cleavage leads to conformational changes in the polymer chain, resulting in swelling or collapse of the polymeric network facilitating the drug release process. Therefore, the ionization of the amino groups in the imidazole rings of histidine is pH controllable, and is defined by its pKa of ~6.10. 

For that reason, Poly(L-histidine) is a promising pH-sensitive polymer with excellent biodegradability, biocompatibility, and fusogenic activity [286]. In fact, when the environment is below pH 6, PLH turns positively charged and hydrophilic, causing the rupture of the endosomal membrane and facilitating the endosomal escape via the so-called proton sponge effect [287,288]. Thus, the protonation of the imidazole groups in PLH when the pH value is below its pKb gives rise a solubility change. However, the stability of the core could be affected by the high sensitiveness to acid pH, and PLH is often used in combination with more hydrophobic polymers, yielding stable copolymers or micelles to coat MSNs. It is important to note that the chain length of PLH affects the antitumoral efficacy of nanoparticles and the pH-responsive drug release rate [289].

**Figure 16:**
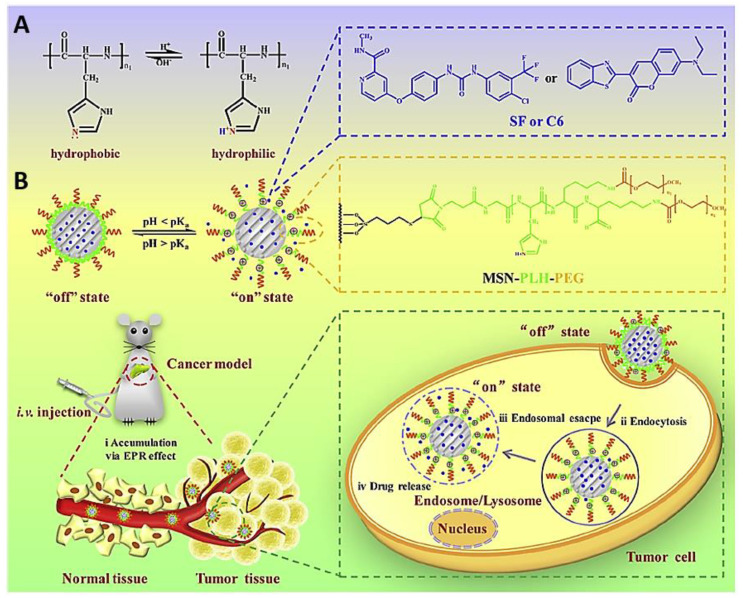
Schematic illustration of: (**A**) The pH-responsive principle of PLH. At neutral pHs, PLH are hydrophobic; whereas at low pH, unsaturated nitrogen in imidazole accepts protons, thereby, PLH displays hydrophilicity. (**B**) Schematic of the pH-controlled drug delivery of SF/MSNs-PLH-PEG bearing an ‘‘on-off” switch. (i) Nanoparticles with an ‘‘off” switch accumulate in tumor site via EPR effect; (ii) PLH accepts proton and facilitates the endocytosis of nanoparticles by tumor cells; (iii) After being endocytosed by tumor cells, the nanoparticles escape from the endosomes/lysosomes, due to the proton sponge effect of PLH; hence, the PLH coating could be switched ‘‘on” gradually; (iv) Drug release from nanoparticles to play its anticancer roles. Adapted from ref. [162]. Copyright 2017, Elsevier Ltd.

Mu et al. provided a promising pH controlled smart system for tumor specific drug delivery based on poly(L-histidine) and poly(ethylene glycol) coated mesoporous silica nanoparticles (MSNs-PLH-PEG) where PLH was grafted to the surface of MSNs [162]. Once MSNs-PLH-PEG have been endocytosed by cancer cells, the “on-state” of PLH coating could be gradually activated in response to acidic endosomes/lysosomes microenvironment and the drug entrapped, sorafenib (SF), could be efficiently released from MSNs-PLH-PEG (Figure 16). This controlled-release is given thanks to the capacity of PLH to changes its solubility at different pH values (7.4–5.0). Moreover, excellent biological results from in vitro cell cytotoxicity and in vivo antitumor studies were obtained, since sorafenib loaded MSNs-PLH-PEG exhibited good anti-proliferation and tumor growth inhibition effects and a negligible hemolytic activity.

Other interesting examples show multifunctional polymeric nanoparticle systems where mesoporous silica core was coated with multi-block polymers for designing smart responsive nanocarriers for simultaneous stimuli [128]. For instance, Demirel et al. designed pH- and redox-triggered magnetic lipid–polymer hybrid nanoparticles (MHNPs) with a core–shell structure. The coating selected for MSNs was a biocompatible and pH-/redox-responsive shell composed by the poly(L-histidine)–poly(ethylene glycol)–lipoic acid polymer that exhibited excellent DOX release profile within 24 h and effective cytotoxicity when it was exposed to an endolysosomal pH of 5.5 and 10 mM GSH. Additionally, this hybrid system showed that the cell viability decreased through apoptosis against breast cancer cell lines [126].

#### 4.3.3. Curcumin and Poly(curcumin) Derivatives

Curcumin is a natural polyphenol extracted from the perennial herb *Curcuma longa* (commonly known as Turmeric), and it presents a very broad antiproliferative activity applied in the prevention of many kinds of cancers [290,291,292,293].

As depicted in Figure 13, the curcumin is a lipophilic fluorescent molecule with phenolic groups and conjugated double bonds, so it has intrinsic fluorescence property [294]. As a consequence, in literature, it is mainly used as an active fluorescent anticancer drug. Nevertheless, it presents poor water solubility, and for that reason, a Pluronic polymer, F127, is usually combined in the coating process to functionalize out the surface of MSNs [295].

Several studies have been reported in the literature in which curcumin plays a dual role as a theranostic and therapeutic agent [164,186]. However, in all of them, curcumin is used as a starting ingredient to develop other curcumin-derivative polymers or even micelles that really act as imaging and capping agents.

In two studies, Xu et al. developed curcumin gatekeepers to close the pores by dense coating. The coating process consists of anchoring the polymer to the surface of MSNs via thiolene ‘‘click” chemistry. On one hand, they used a bis-acrylated curcumin together with a Pluronic polymer, F1127, that improves the dispersity of the nanoparticles into tumoral microenvironment [296]. On the other hand, they performed a curcumin polymer shell gated MSN synthesized from of curcumin polymer by a facile one-pot method [163].

**Figure 17:**
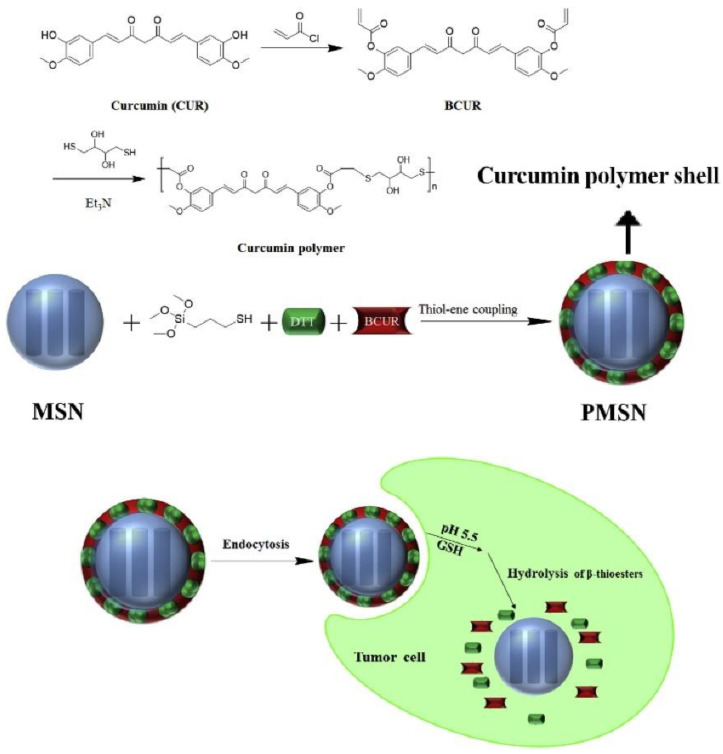
Illustration for the preparation of curcumin polymer, PMSN and intracellular microenvironment triggered release. Reproduced with permission from ref. [163]. Copyright 2018 Elsevier Inc.

In both studies the responsive release appeared in presence of GSH and acidic microenvironment pH (5.5). In these conditions, the curcumin coating was removed to induce the drug release intracellularly through the hydrolysis of β-thioesters, triggered by these endogenous factors (Figure 17).

## 5. Conclusions and Future Perspectives

The application of natural biopolymers to control the encapsulation and release of different therapeutic molecules from silica-based mesoporous nanoparticles in response to internal stimuli has here been reviewed. 

The effort to replace synthetic materials with alternatives of natural origin is of great relevance in the field of nanomedicine since the intrinsic biocompatibility, low toxicity, and low immunogenicity of natural biopolymers is one of the main reasons for their extensive investigation. In addition, the composition of biopolymers as well as their biodegradability ensure their structural and chemical shift in response to specific stimuli of pathological environments. For these reasons, multiple approaches have shown the rational use of biopolymers as MSNs coatings. 

A large number of in vitro and in vivo preclinical studies have reported their promising application as gatekeeper agents. These entities provide an effective and biocompatible barrier against premature leakage of therapeutic molecules from mesopores while ensuring controlled release upon specific endogenous stimuli. Several research studies have also reported the capacity of natural biopolymers for increasing cellular uptake and they kill malignant cells with a high selectivity. Recent studies also evaluated the combination of several types of polymers to develop novel nanocarriers with enhanced drug delivery performances. In these multi-polymeric systems, each polymeric motif plays a different role such as coating, targeting ligand, or even linkers to form a shell. The use of specifical ratios of different classes of moieties permits to harness the principal features of each one of them and thus to develop more intelligent and effective nanocarriers able to respond to more than one stimulus simultaneously offering a local and precise treatment of the diseased tissue.

It is worth to mention that there are still open issues related with both MSNs core and biopolymer coatings that need to be addressed for the effective translation of these nanosystems. The production of MSNs on a large scale with appropriate reproducibility and control over synthetic parameters, such as size and porosity that influence release profiles, needs to be implemented and regulated. Moreover, the efficacy and safety of MSNs in vitro or in small animal models have been object of comprehensive studies, but their evaluation in more complex animal models or humans is still in its early stages. Similarly, the biocompatibility, biodegradability, and in vivo fate of different biopolymers as single components and as part of smart MSNs-based nanosystems need to be thoroughly evaluated. Similarly, the modification of MSNs physicochemical features after the biopolymeric coatings should be carefully considered. For instance, aggregation of coated nanoparticles due to the crosslinking effect of biopolymeric macromolecules has been frequently reported and has to be addressed through the optimization of coating procedures to lead to effective applications.

Although there is certainly still much to be done, continuous investigations are carried out to establish smart biopolymer coatings as a valuable approach to overcome non-specific release, and this could constitute a promising line of investigation to facilitate MSNs clinical translation, providing powerful tools to get closer to the patient’s health.

## Figures and Tables

**Table 1:** Representative examples of MSNs coated with natural biopolymers responsive to endogenous stimuli for different drug delivery applications.

*Polysaccharides*							
Biopolymer		Endogenous Stimulus	Silica Core	Cargo	Targeting Ligand	Application	Ref.
Chitosan		pH	Hollow MSNs	Doxorubicin	-	Cancer	[137]
		pH	MCM-41	Curcumin	-	Glioblastoma	[138]
		pH	MCM-41	Doxorubicin and antimiR-21	AS1411 aptamer	Colorectal and breast cancer	[139]
		pH and redox	Mn-doped MSNs	Doxorubicin and Tariquidar	Folic acid and hyaluronic acid	Drug resistant and cancer stem cells	[140]
		enzymatic	Hollow MSNs	Doxorubicin	-	Cancer	[141]
		pH	MCM-41	Dexamethasone and BMP-2	-	In vivo bone regeneration	[142]
Alginate		pH and redox	MCM-41	Doxorubicin	-	Cancer	[143]
Alginate/ Chitosan		pH	MCM-41	Doxorubicin	-	Cancer	[144]
Hyaluronic acid		pH	Fe_3_O_4_NPs @MSNs	Doxorubicin	-	Targeted treatment of breast cancer	[145]
		enzymatic	MCM-41	5-fluorouracil	-	Targeted treatment of colon cancer	[146]
		enzymatic	MCM-41	Simvastatin	-	Atherosclerosis	[147]
		Enzymatic and pH	MCM-41	Rosuvastatin	CD9	Atherosclerosis	[148]
		enzymatic	PLNPs ^a^ @MSNs	Cinnamaldehyde	-	Multidrug resistant antibacterial infections	[149]
Cellulose		Enzymatic and pH	MCM-41	Doxorubicin	-	Cancer	[150]
Carboxy methyl cellulose		pH	Hollow MSNs	Doxorubicin	AS1411 aptamer	Colorectal and breast cancer	[151]
β-cyclodextrin		pH and redox	MSNanorods	Doxorubicin	Hyaluronic acid	Cancer	[152]
		redox	Hollow MSNs	Glabridin	Ada-R8	Transdermal delivery	[153]
***Protein-based biopolymers***							
**Biopolymer**		**Endogenous stimulus**	**Silica core**	**Cargo**	**Targeting** **ligand**	**Application**	**Ref.**
Silk Fibroin		pH	MCM-41	Tioguanine (TG)	-	Leukemia	[154]
Gelatin		pH	MCM-41	Topotecan (TOP)	Folic Acid (FA)	Cancer	[155]
		Enzymatic (MMP-2)	MCM-41	Doxorubicin	Folic Acid (FA)	Cancer	[103]
		Bienzymatic (MMP-2 and Hyaluronidase)	MCM-41	Doxorubicin	Hyaluronic Acid (HA)	Breast cancer	[156]
Albumin (HSA ^b^ and/or BSA ^c^)		redox	MCM-41	Epirubicin (EPI)	Folic acid	Cancer	[157]
		pH	MCM-41	Gemcitabine	-	Cancer	[158]
Polydopamine		pH	MCM-41	Doxorubicin	-	Cancer	[159]
		pH	MCM-41	Anderson-type polyoxomolybdate	Glucosamine	Breast Cancer	[160]
		pH	MCM-41	Desipramine (DES)	-	Cancer	[161]
Poly(L-histidine)		pH	MCM-41	Sorafenib	-	Cancer	[162]
PCUR ^d^	BCUR ^e^	Enzymatic (GSH)	MCM-41	Doxorubicin	-	Cancer-therapeutic imaging	[163]
	CUR ^f^	pH	MCM-41	Doxorubicin and Curcumin	-	Cancer	[164]

*^a^ PLNPs: Persistent Luminescent Nanoparticles; ^b^ HSA: Human Serum Albumin; ^c^ BSA: Bovine Serum Albumin; ^d^ PCUR: Polycurcumin; ^e^ BCUR: Bis-acrylate terminated curcumin (curcumin polymer); ^f^ CUR: Curcumin.*

## Data Availability

Not applicable.
